# Utility of constructed wetlands for treatment of hospital effluent and antibiotic resistant bacteria in resource limited settings: A case study in Ujjain, India

**DOI:** 10.1002/wer.10783

**Published:** 2022-09-08

**Authors:** Vivek Parashar, Surya Singh, Manju R. Purohit, Ashok J. Tamhankar, Dharmpal Singh, Madhanraj Kalyanasundaram, Cecilia Stålsby Lundborg, Vishal Diwan

**Affiliations:** ^1^ R.D. Gardi Medical College Ujjain India; ^2^ Division of Environmental Monitoring and Exposure Assessment (Water & Soil) ICMR – National Institute for Research in Environmental Health Bhopal India; ^3^ Department of Global Public Health Karolinska Institutet Stockholm Sweden; ^4^ ICMR – National Institute of Epidemiology Chennai India

**Keywords:** antibiotic resistant bacteria, constructed wetlands, *Phragmites*, *Typha*, wastewater

## Abstract

Increasing generation of wastewater and its indiscriminate disposal is detrimental to human and animal health. Resource‐limited settings often struggle for efficient wastewater treatment systems owing to lack of funds and operational difficulties. Therefore, alternative treatment systems involving low expenditure and simplistic operations are need of the hour. Constructed wetlands are one such alternative that can efficiently remove variety of pollutants from wastewater. In this study, we have assessed the utility of constructed wetlands for treatment of hospital wastewater in Ujjain. An in‐house wetland system was designed and constructed using *Typha latifolia* and *Phragmites karka* . Results showed that wetland was efficient for removal of various physico‐chemical and biological contaminants, namely, biochemical‐oxygen‐demand (77.1%), chemical‐oxygen‐demand (64.9%), turbidity (68.3%), suspended‐solids (63%), total‐phosphorus (58.7%), nitrate‐nitrogen (33%), fecal coliforms (96.8%), and total coliforms (95.6%). Paired *t* test revealed that removal efficiencies for various parameters were significantly different among *Phragmites*, *Typha*, and control cells (*p* ≤ 0.05). Study also depicted that most of the bacterial isolates in inlet wastewater were selectively resistant to antibiotics (ciprofloxacin and sulphamethaxazole) as well and these isolates were also removed. Precisely, *Typha* was fairly suitable for antibiotic resistant bacteria removal. Thus, constructed wetlands were found to be one of the suitable options for wastewater treatment in resource‐limited settings.

**Practitioner Points:**

- Constructed wetlands are one of the suitable options for wastewater treatment in resource limited settings.
- These systems involve wetland vegetation, soil, and associated microbial assemblages to improve the water quality.
- *Typha* and *Phragmites* were found to be efficient for treating the hospital wastewater.
- Experiments showed that antibiotic resistant bacteria may also be removed through constructed wetland systems.
- Easy operation, cost effectiveness, and efficiency are important attributes.

## Graphical abstract

Graphical abstract shows the inlet wastewater received from a hospital (study setting) in Ujjain, which was then fed to the constructed wetland (CW) unit. Wastewater was stored for some time in storage tank, and then, it was passed through three different cells, namely, *Typha latifolia* cell, *Phragmites karka* cell, and control cells. The resultant removal efficiency for various water quality parameters is shown in the figure.

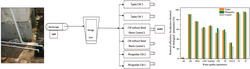

## INTRODUCTION

The wastewater generated out of various domestic, commercial, and industrial activities may contain various physical, chemical, and biological moieties, many of which are harmful to environment, animals, and human beings. In order to maintain the quality of limited freshwater resources available, it is essential to treat the generated wastewater to a certain standard before it is discharged into surface/ground water bodies (Jain et al., 2019, 2021). However, as per the recent data, there exists a gap of approximately 63% between the “amount of sewage generated” and the “operational sewage treatment capacity in India” (CPCB, 2021). Thus, the huge amount of untreated wastewater is ultimately discharged into the surface water sources, making it a sink of various physico‐chemical pollutants and microorganisms (Pazda et al., 2019; Singh et al., 2022; Singh, Kalyanasundaram, & Diwan, 2021; UNWWDR, 2017). Many a times, the untreated and treated wastewater also contains traces of various pharmaceutical compounds, such as antibiotics, unused drugs, or the metabolites of drugs (Diwan et al., 2010; Rizzo et al., 2013). These residues contribute to the development of antibiotic resistance in organisms in the long term (Diwan et al., 2017; Hanna et al., 2020; Pazda et al., 2019; Rizzo et al., 2013). Presence of drugs/antibiotic resistant microorganisms in the water resources poses a serious threat to the community owing to transmissible genes and their better survival potential in wide range of environmental conditions (Larsson, 2014; WHO, 2020). Antibiotic resistant microorganisms are evolving faster than earlier anticipated, leading to the situation, where antimicrobial medicines will stop functioning against the same microbes for which they were found efficacious earlier (WHO, 2020). This may create an alarming situation. Removal of these resistant organisms from the wastewater and modifying the physico‐chemical and biological contaminants is therefore essential for restricting their further growth and transmission (Singer et al., 2016). Therefore, considering the complexity and amount of wastewater generated, various treatment options have been made available by the researchers. However, maintenance and operational costs of running a treatment plant are the major deciding factors in resource limited settings. In such a scenario, constructed wetlands may provide a suitable alternative to treat the wastewater.

Constructed wetlands are the water treatment systems that use natural processes involving wetland vegetation, soils, and their associated microbial assemblages to improve the water quality (Barancheshme & Munir, 2018; EPA, 2017). These units have gained attention owing to their easy operation, cost effectiveness, and efficiency (Datta et al., 2016; Fang et al., 2017; Tilak et al., 2017). Successful application of constructed wetlands has been reported for wastewater treatment, heavy metals removal, and antibiotic resistant genes removal using various plant species, such as *Canna*, *Eichhornia*, *Phragmites*, *Typha*, and *Ageratum* (Alvarez et al., 2017; Barancheshme & Munir, 2018; Jamwal et al., 2021; Juwarkar et al., 1995; Kumar et al., 2016; Rampuria et al., 2021; Rana et al., 2011; Tilak et al., 2017). In the present study, the utility of constructed wetlands was assessed for the treatment of wastewater generated from a hospital in Ujjain.

## MATERIALS AND METHODS

### Setting

The study was conducted in Ujjain city of Central Indian Province of Madhya Pradesh. Ujjain is a small town having a population of 0.52 million with approximately 60% residing in semi‐urban areas (Census, 2011; Singh, Parashar, et al., 2021). More specifically, the setup was in the Medical College Campus of the city located in a rural area, 6 km from the city. During the study period, the hospital had approximately 720 beds, and the total number of patients per day ranged from 1,400 to 1,600. The total wastewater generated from the hospital facility was approximately 150 kiloliters per day (KLD).

The average air temperature during the study period was 29.67°C, with maximum and minimum temperatures of 34.47°C and 22.60°C, respectively. A total of 1,238.88 mm rainfall was recorded during the study period. The average air temperature on specific sampling days was 28.96°C, while average maximum and minimum temperature were 34°C and 21°C, respectively. During summer, the average temperature was recorded as 32.3°C, while during winters and rains, the temperature was recorded as 23.23°C and 31.35°C, respectively.

### Experimental set‐up

**FIGURE 1:**
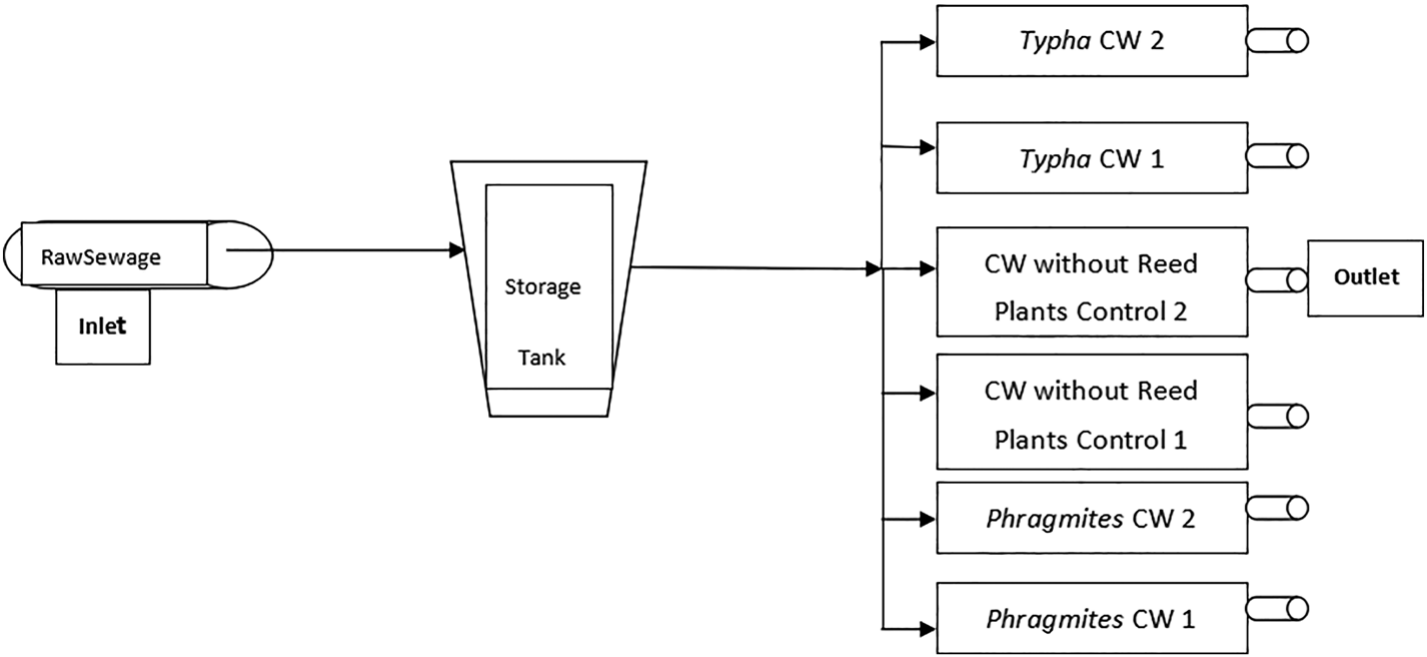
Schematic of the designed constructed wetland (CW)

A demonstration level horizontal subsurface constructed wetland was designed and set‐up in the medical college campus of Ujjain. Schematic of the wetland is shown in Figure 1. Sand and gravel were used as media. *Phragmites karka* and *Typha latifolia* were planted as the main vegetation in the wetland cells.

A polyvinylchloride storage tank of 1,000 L was placed prior to the constructed wetland cells to act as the storage and sedimentation cell (Figures 1 and S1a,b). Six horizontal subsurface flow experimental wetland cells (1.524 m × 0.635 m × 0.508 m) were constructed. All the six cells were filled up to 0.47 m with alluvial washed river gravel and sand (approximately 10–15 mm diameter, porosity 35–40%) in order to reduce the surface compaction (Kadlec & Knight, 1996; Tanner et al., 1995). Further, gravel (30 mm) was placed at the inlet and outlet of each cell to provide stability to the inner media. It also helped in reducing the total suspended solids (TSS). The salient features of the constructed wetland are shown in Table 1.

**TABLE 1:** Salient features of experimental constructed wetland cells

Design type	Horizontal subsurface flow
Length (m)	1.524
Width (m)	0.635
Depth (m)	0.508
Area (m^2^)	0.967
Discharge (m^3^/day)	0.144
Hydraulic loading rate (m/day)	1.016
Retention time (day)	1.19
Porosity (%)	35–40
Plant density (in 0.967 m^2^)	15

Water level in the wetland was maintained at 0.05 m. below the gravel surface owing to the subsurface flow design. Outflow water volume was also monitored. For plantation, the saplings and roots of *Typha latifolia* and *Phragmites karka* were collected from the local ponds and river banks. Out of the six wetland cells, two cells were planted with *Typha* (Figure S1c) and next two with *Phragmites* (Figure S1d). The other two cells were kept unplanted (to act as control) (Figure 1). These cells were initially filled with the tap water and allowed for the acclimatization of plants up to 10–20 days. Later, fresh wastewater was added to all the wetland cells.

### Sampling

Samples were collected from inlet and outlet of each constructed wetland cells. Collection and preservation were done as per the American Public Health Association (APHA) guidelines (APHA, 2005). This study was conducted from October 2015 to September 2017. A total of seven samples were collected from the study site in each batch of sampling—one sample each from storage cell and other six wetland cells in the months of summer (S), rains (R), and winter (W). Samples were also collected in the month of autumn; however, the number of samples was insufficient; therefore, samples of winter and autumn months were combined.

### Sample analysis

The collected influent and effluent samples were tested for various physico‐chemical (pH, turbidity, biochemical oxygen demand [BOD], total suspended solids [TSS], chemical oxygen demand [COD], total phosphorous [TP], and nitrate–nitrogen [NO_3_‐N]) and biological parameters (fecal coliforms [FC] and total coliforms [TC]) using standard methods (APHA, 2005).

For bacterial analysis, tenfold serial dilution (1:100, 1:1,000 as per turbidity of sample) of surface water was done in 0.9% normal saline. The diluted samples were filtered through standard membrane filtration technique using nylon membrane filters (47 mm, 0.45 μ) for a minimum of 2.5 h. The membrane was taken out of assembly to place on selective and differential media, HiChrome Coliform Agar, to culture coliforms for 24 h with incubation at 37°C. Bacterial enumerations were done to estimate total coliform count and total *Escherichia coli* (*E. coli*) count in CFU/100 ml on agar.

Antibiotic susceptibility testing was simultaneously performed by Kirby Bauer disk diffusion test (CLSI, 2014) on Muller Hinton (MH) Agar for two different antibiotics, namely, ciprofloxacin (CPF) and sulphamethaxazole (SMX), with the bacterial suspension of 0.5 McF turbidity. Antibiotic discs were incubated for 24 h on bacterial lawn to read the zone of inhibition. The zone diameter was measured and analyzed as per Clinical and Laboratory Standard Institute Guidelines (CLSI, 2014; Magiorakos et al., 2012).

### Data analysis

Data obtained after the wastewater sample analysis were used to evaluate the removal efficiency and other comparisons among the wetland cells. Removal efficiency was calculated for each parameter using the concentration of a specific parameter in the inlet (*In*) and outlet (*Out*), as shown in Equation (1).

(1) $$\text{Removal efficiency}=\frac{\text{In}-\text{Out}}{\text{In}}\times 100$$

The results were compared among the *Typha*, *Phragmites*, and control cells. Further, statistical analysis was done using SPSS software. Mean and standard deviations were calculated. Significant change in mean values of the parameters, namely, BOD, COD, turbidity, phosphorous, NO_3_‐N, pH, and TDS, between raw sewage and the experimental cells (*Typha*, *Phragmites*, and control cells—within group change) was tested by paired *t* test. Non‐parametric test (Wilcoxon signed rank test) was used for the skewed variables. The change in mean values of the parameters between the groups was assessed by using one‐way ANOVA with posthoc Bonferoni correction.

## RESULTS

### Characteristics of the water with respect to physicochemical parameters before and after the treatment

**FIGURE 2:**
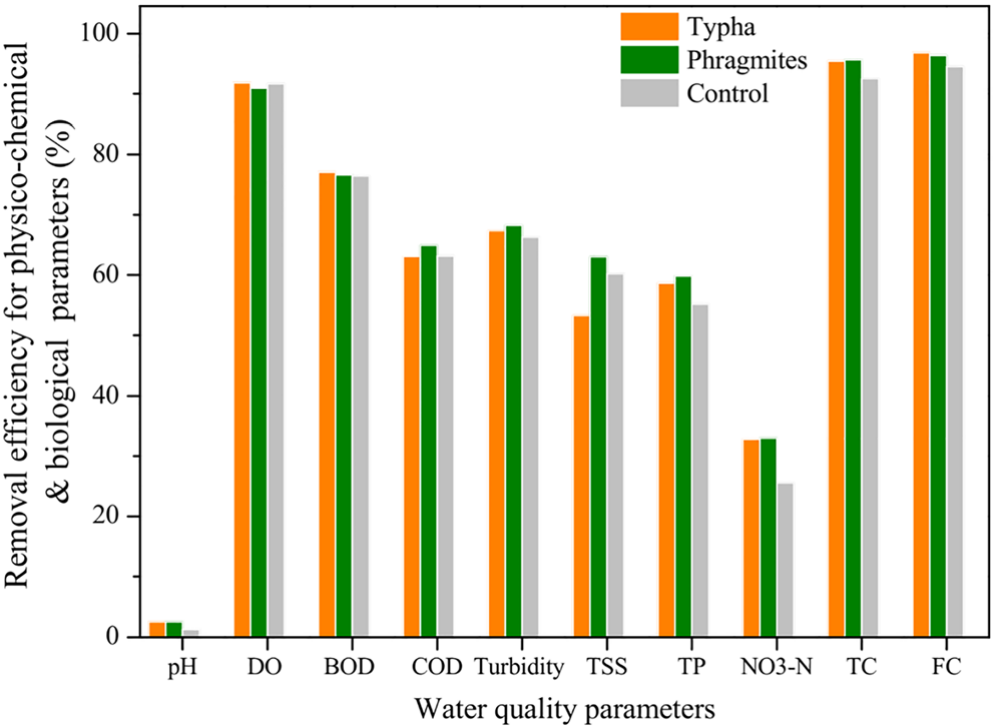
Removal efficiency of constructed wetland cells for physico‐chemical and biological parameters

Various parameters of the water quality in the influent stream (i.e., wastewater) and effluent stream (i.e., treated water) are shown in Table S1. Results show that all the parameters were improved in effluent stream as compared to the influent stream. The pollutant removal efficiency of the wetland cells packed with different plantations is shown in Figure 2. It can be seen that the highest removal efficiency for BOD and FC is of the *Typha* cells while *Phragmites* cells are comparatively more efficient for the removal of COD, turbidity, TSS, TP, NO_3_‐N, and TC.

Descriptive statistics and its significant values for different parameters (paired *t* test) revealed that pH removal efficiencies of *Phragmites*, *Typha*, and control cells were significantly not different (*p* > 0.05). However, removal efficiencies for BOD, COD, turbidity, TSS, total phosphorus, and nitrate–nitrogen were significantly different among the *Phragmites*, *Typha*, and control cells (*p* ≤ 0.05). Significant difference between the cells after treatment was not found.

### Seasonal variation in the effluent water quality with respect to physicochemical parameters

**FIGURE 3:**
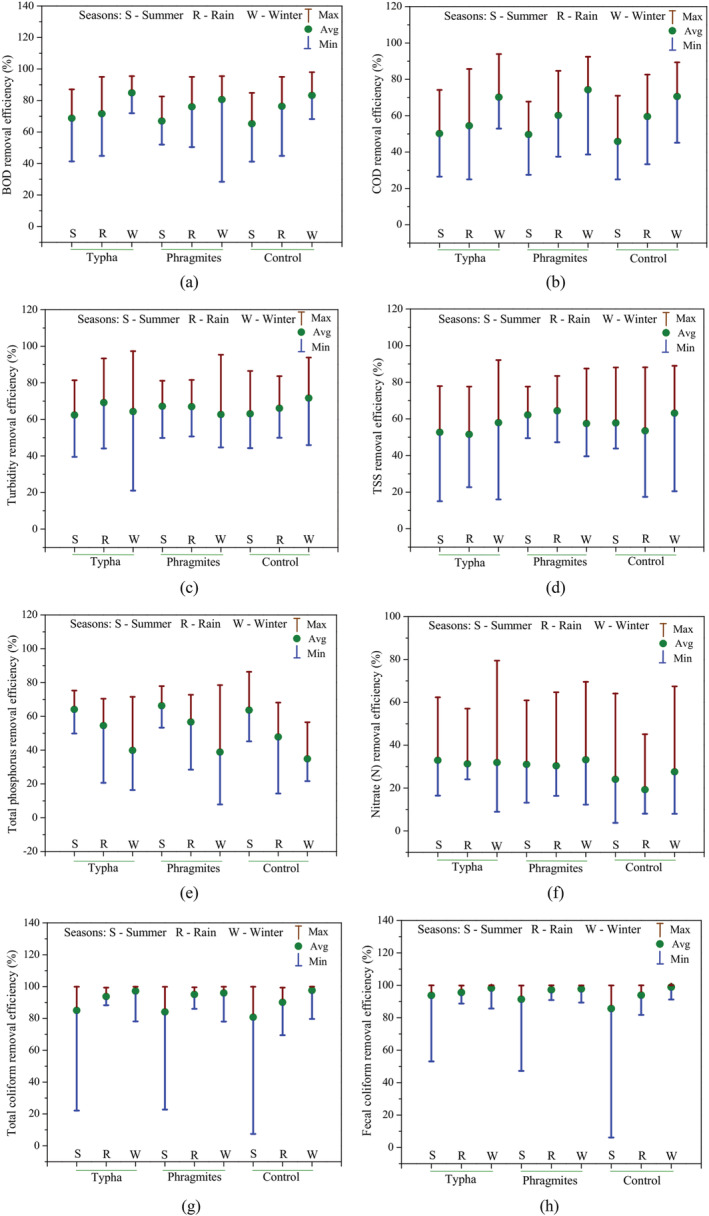
Seasonal variation in the removal efficacy of pollutants by different constructed wetland cells: (a) BOD, (b) COD, (c) turbidity, (d) TSS, (e) TP, (f) NO_3_‐N, (g) total coliforms, and (h) fecal coliforms

The seasonal effect on pollutant removal efficiency for different wetland cells is shown in Figure 3a–h. For BOD and COD removal, efficiency is the highest during winter season in all the wetland cells (Figure 3a,b); however, there is mixed pattern among seasons for turbidity and TSS removal in the wetland cells (Figure 3c,d). For total phosphorous, the removal efficiency is the highest in summer season (Figure 3e). In case of total coliforms and fecal coliforms (Figure 3g,h), it can be seen that removal efficiency is the lowest in summer season in all the wetland cells.

It is further inferred that TSS removal percentage among *Phragmites*, *Typha*, and control wetland cells was different. In summer and rainy season, the *Phragmites* wetland cells were having higher removal percentage compared to control and *Typha* cells while in winter season, control wetland cells were having the higher removal percentage than *Phragmites* and *Typha* (Figure 3d). In case of NO_3_‐N also, removal percentage among *Phragmites*, *Typha*, and control wetland cells was different in different seasons. *Phragmites* and *Typha* cells were having higher removal percentage than control in all the seasons. Further, control wetland cells were having lower removal efficiency in rainy season compared to summer and winter (Figure 3f).

### Characteristics of the water with respect to antibiotic resistance bacteria before and after the treatment and seasonal variation therein

**FIGURE 4:**
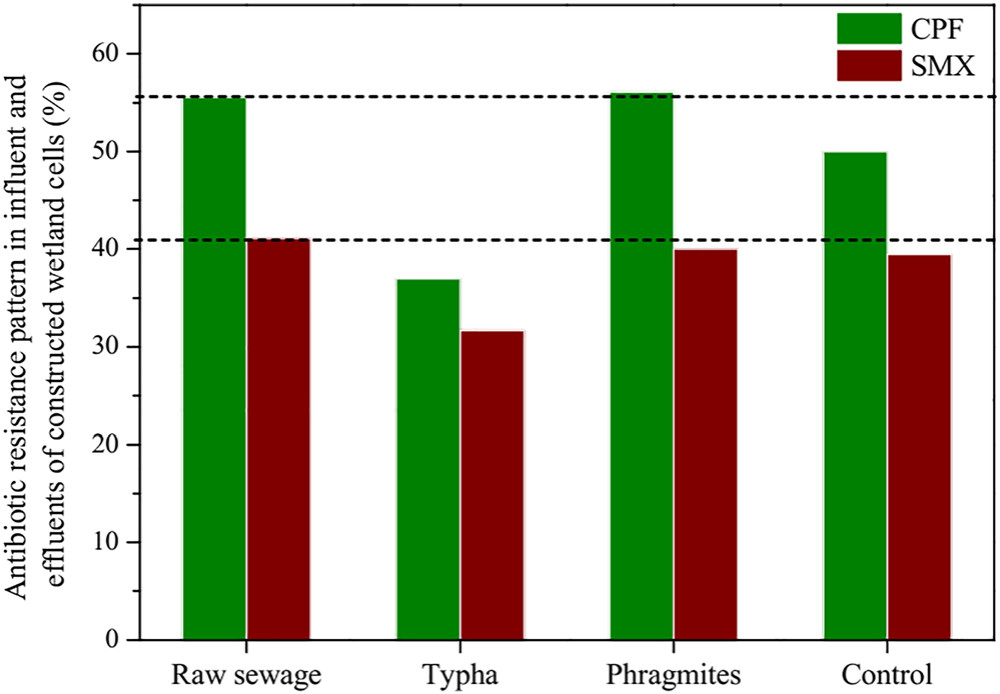
Antibiotic resistance pattern (%) of *E. coli* isolates for ciprofloxacin (CPF) and sulphamethaxazole (SMX) in influent (raw sewage) and effluents of constructed wetland cells (dotted lines in the graph indicate the antibiotic resistance pattern [%] in the raw sewage)

Among the *E. coli* isolates from influent, 55.6% isolates were found to be resistant to ciprofloxacin (CPF), and 41.1% isolates were found to be resistant to sulphamethaxazole (SMX) (Figure 4). As this influent was introduced into the different cells of constructed wetlands, a decrease in the concentration of antibiotic resistant bacteria was seen in the effluent water. *Typha* cells were able to reduce the amount of CPF resistant bacteria up to 37% and up to 31.67% for SMX resistant bacteria (Figure 4). After passing the influent through control cells, the amount of CPF resistant bacteria came down to 50% and SMX resistant bacteria to 39.44% (Figure 4). *Phragmites* cells did not contribute much to removal; rather, there was increase in the concentration of CPF resistant bacteria after passing through *Phragmites* cells. It might happen sometimes because of the favorable growth conditions for the bacteria provided by the wetland cells. Nevertheless, the results indicated that constructed wetland cells are able to remove the antibiotic resistant bacteria.

**FIGURE 5:**
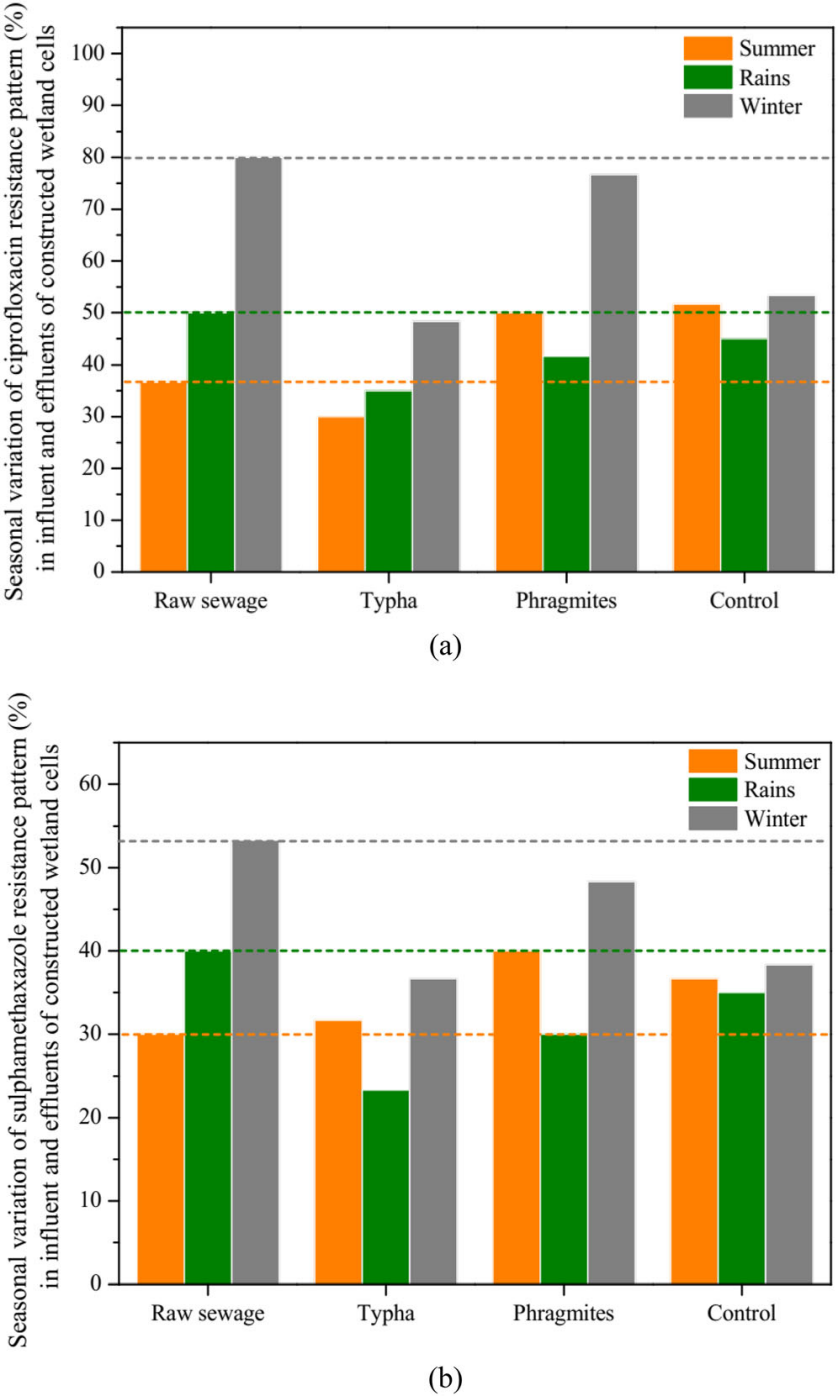
Seasonal variation in antibiotic resistance pattern (%) of *E. coli* isolates for ciprofloxacin (CPF) (a) and sulphamethaxazole (SMX) (b) in influent (raw sewage) and effluents of constructed wetland cells (dotted lines in the graph indicate the antibiotic resistance pattern [%] in the raw sewage in different seasons)

It was also found that in case of *Typha* cells, removal of CPF resistant *E. coli* isolates was highest in the winter season and minimum in the summer season (Figure 5a). It might happen due to better survival and proliferation conditions available in the summer season for bacteria, as compared to winters. Similarly, in case of SMX resistant *E. coli* isolates, highest removal was achieved in the months of winter followed by rains. Moreover, the removal was more in the cells of *Typha* and control while least in the cells of *Phragmites* (Figure 5b).

## DISCUSSION

The results in this study showed that constructed wetlands could be a promising technology for the removal of physico‐chemical/biological pollutants and antibiotic resistant bacteria. Moreover, understanding the seasonal variation in the removal process is also crucial while planning the overall wastewater management (Diwan et al., 2013). There has been some debate on the importance of plants in coliforms and antibiotic‐resistant bacteria removal by constructed wetland systems. Investigations have shown that wetland systems with vegetation have higher efficiency for the total, fecal, and antibiotic resistant bacteria removal, compared to un‐vegetated wetlands (Lekeufack et al., 2017; Martin et al., 2012). Results of the present study are found in conformity with the above findings, as the study reveals that higher removal of physico‐chemical pollutants and antibiotic resistant bacteria took place in the vegetated wetlands than in the un‐vegetated (control) wetland systems (Figures 2 and 4). The reason for the better efficiency of vegetated wetlands is that plants enhance the microbial density as well as activity, particularly on the root surface due to the root oxygen release. This enhances microbial activity, which helps to degrade the chemical moieties and bacteria present in the wastewater (Bôto et al., 2016). Effects of plant growth on the removal of various pollutants have also been seen, where it was found that increasing plant growth provides better filtration medium along with enhancing the surface area and food opportunities for microorganisms, thus resulting in better removal efficiency (Karathanasis et al., 2003). Moreover, efficiency of the vegetated wetland system can also be increased by growing more than one plants together (Karathanasis et al., 2003). Further, sedimentation/sorption onto soil or wetland media, biodegradation, photo‐degradation, hydrolytic action, and plant uptake are other mechanisms that help to clean the water (Liu et al., 2013). Natural die‐off of bacteria due to competition with the consortium of organisms and toxins released from plants and other microorganisms also help in the removal of microbial contamination (Bôto et al., 2016; Karimi et al., 2014).

Both the two types of vegetated wetland cells employed in the present study, namely, *Typha latifolia* and *Phragmites karka*, showed significant capability to treat hospital effluent. Microbiological contaminants (FC and TC) could be removed by more than 95%, while efficiency for the removal of physico‐chemical parameters was ranged from 32–77%. These findings also match with the study reported by Datta et al. (2016) in which *Typha* vegetation was found to result in steady removal efficiency of 35–40% for ammonical nitrogen, though in combination with water hyacinth or water lettuce. Similar kind of study was also carried out for the treatment of municipal wastewater in Ujjain city (India) using *P. karka* (Billore et al., 1999). This study reported the removal efficiencies as 78% for ammonical nitrogen and TSS and 58–65% for phosphorus, BOD, and TKN (Billore et al., 1999). Angassa et al. (2019) also carried out the investigations using *P. karka* in subsurface flow constructed wetland cells for the treatment of municipal wastewater. It was reported that removal efficiency for COD, total nitrogen, and TP were 94%, 97%, and 90%, respectively. Study also showed that removal efficiency was decreased upon increasing the hydraulic loading rates in the wetland system. The removal efficiency reported in the present study is less as compared to the Angassa et al. (2019) However, the differences obtained in removal efficiency may be attributed to the hydraulic loading rate. The hydraulic loading rate of 1.016 m/day in the present study is significantly higher than 0.025 m/day in Angassa et al. (2019) resulting in comparatively lower efficiency.

Moreover, in the present study, *Typha* cells showed better efficiency to treat not only the physico‐chemical parameters but also the antibiotic resistant bacteria. The basic reason for the ability of *Typha* to act as a remediator is its response to the oxidative stress posed by various pollutants and antibiotic resistant bacteria (Liu et al., 2019). The stress is a consequence of accumulation of various reactive oxygen species that hinder the normal cellular and biochemical processes in the plant system. Therefore, plant develops an effective anti‐oxidative response as a defense mechanism, by producing enzymes such as superoxide dismutase (SOD), catalase (CAT), and peroxidase (POD) (Xu et al., 2011). This stress coping mechanism of the *Typha* sp. might have contributed to its better efficiency for antibiotic resistant bacteria.

## CONCLUSION

The results of this study indicate that the wetland system, having *Typha* and *Phragmites* vegetation, was able to reduce the concentration of a variety of adverse physico‐chemical parameters along with the coliform bacteria. The highest removal efficiency for BOD (77.1%) and fecal coliforms (96.8%) was found for the *Typha* cells, while *Phragmites* cells were found comparatively more efficient for the removal of COD (64.9%), turbidity (68.3%), TSS (63%), Total Phosphorous (58.7%), NO_3_‐N (33%), and total coliforms (95.6%). Effect of seasonal variation was also seen, and it was found that removal efficiency was varied for various parameters in different seasons. Moreover, the designed constructed wetland, *esp*. the *Typha* cells, was also able to remove the antibiotic resistant bacteria considerably. Thus, it can be concluded that the constructed wetland systems may prove to be one of the suitable options for the treatment of hospital wastewater in resource limited settings, where high end treatment facilities are not available. Nevertheless, low‐cost, repeated use for long time, and easy operational mechanisms and maintenance further enhance the scope of constructed wetland utility.

## CONFLICT OF INTEREST

The authors declare that there are no competing interest.

## AUTHOR CONTRIBUTIONS

The investigation was carried out by Vivek Parashar, Manju R. Purohit, and Vishal Diwan. Methodology of the work was framed by Vivek Parashar, Manju R. Purohit, Ashok J. Tamhankar, Dharmpal Singh, Cecilia Stålsby Lundborg, and Vishal Diwan. Formal analysis was done by Surya Singh, Vivek Parashar, Dharmpal Singh, and Madhanraj Kalyanasundaram. Original draft of the manuscript was written by Surya Singh. Review and editing were done by Surya Singh, Manju R. Purohit, Ashok J. Tamhankar, Madhanraj Kalyanasundaram, Cecilia Stålsby Lundborg, and Vishal Diwan. Supervision was done by Ashok J. Tamhankar and Vishal Diwan. Visualization was done by Dharmpal Singh and Madhanraj Kalyanasundaram. Funding acquisition was done by Vivek Parashar, Cecilia Stålsby Lundborg, and Vishal Diwan, and overall project was administered by Cecilia Stålsby Lundborg and Vishal Diwan. Vivek Parashar and Surya Singh share equal first authorship.

## Supporting information


**Figure S1.** Design of constructed wetland cells (a) Experimental wetland cells at the ground, (b) Experimental wetland cells at the height of 2 feet to avoid flood situation (c) *Typha* sp. plantation, (d) *Phragmites* sp. plantation
**Table S1.** Average values of water quality parameters before and after the treatment in constructed wetland cells

## Data Availability

All the data are included in the paper and its supporting information.
