# Supplementary material for: Utility of constructed wetlands for treatment of hospital effluent and antibiotic resistant bacteria in resource limited settings: A case study in Ujjain, India
Source: Water Environ Res. 2022 Sep 8;94(9):e10783. doi: 10.1002/wer.10783 (PMC9544608; doi:10.1002/wer.10783)
Supplement: Supplementary file 1 — Figure S1. Design of constructed wetland cells (a) Experimental wetland cells at the ground, (b) Experimental wetland cells at the height of 2 feet to avoid flood situation (c) Typha sp. plantation, (d) Phragmites sp. plantation Table S1. Average values of water quality parameters before and after the treatment in constructed wetland cells [file WER-94-0-s001.docx]

**Supplementary Information**

| 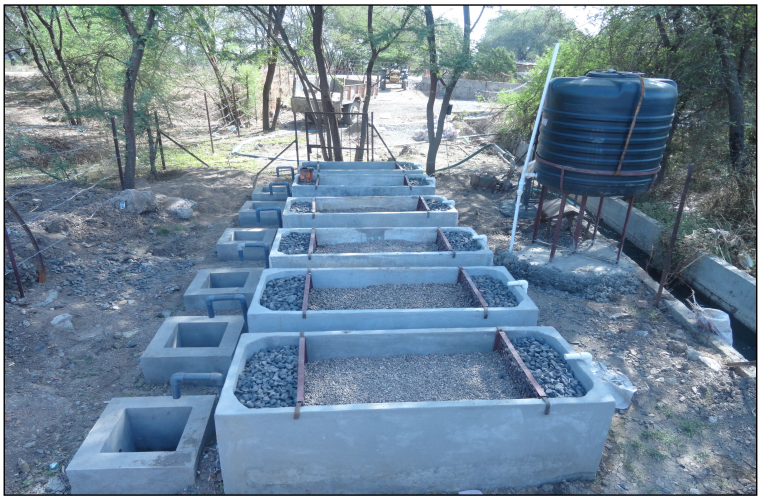 | 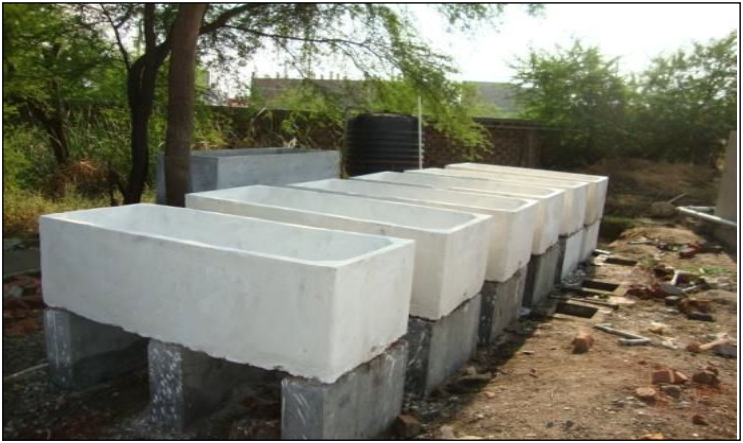 |
| --- | --- |
| (a) | (b) |
| 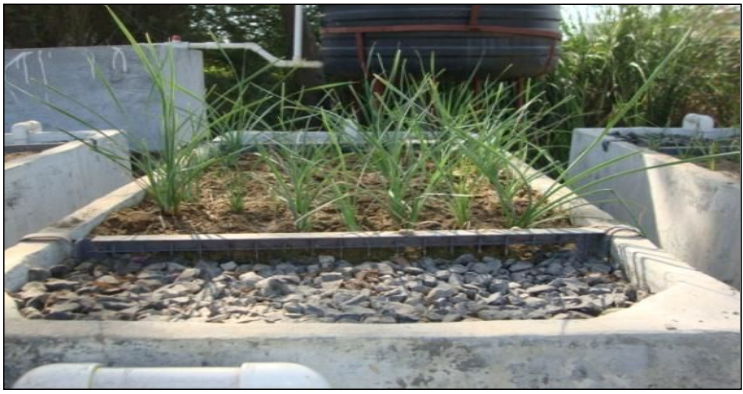 | 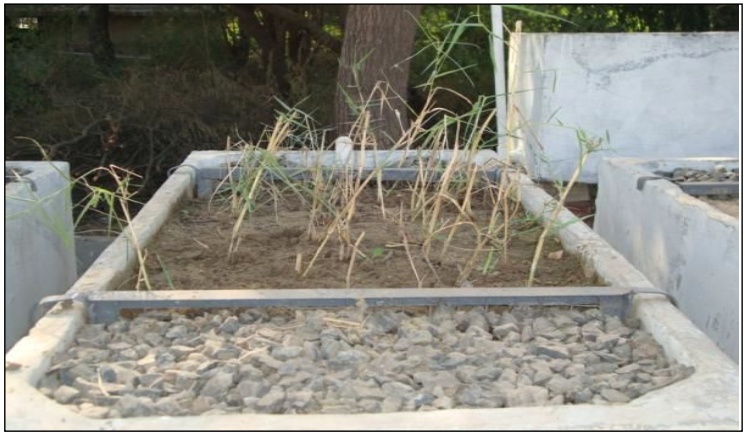 |
| (c) | (d) |
| **Figure S1.** Design of constructed wetland cells (a) Experimental wetland cells at the ground, (b) Experimental wetland cells at the height of 2 feet to avoid flood situation (c) *Typha sp*. plantation, (d) *Phragmites sp.* plantation | |

**Table S1.** Average values of water quality parameters before and after the treatment in constructed wetland cells

| \| **Water quality parameters** \| \| **Type of constructed wetland cells** \| \| \| \| --- \| --- \| --- \| --- \| --- \| \| ***Typha* cell** \| ***Phragmites* cell** \| **Control** \| \| pH \| In* (SD) \| 7.7 (0.26) \| \| \| \| Out* (SD) \| 7.5 (0.36) \| 7.6 (0.36) \| 7.7 (0.32) \| \| *p*-value \| 0.07 \| 0.25 \| 0.97 \| \| BOD (mg/L) \| In (SD) \| 136.32 (49.52) \| \| \| \| Out (SD) \| 32.92 (25.78) \| 31.85 (17.80) \| 32.12 (21.33) \| \| *p*-value \| 0.001 \| 0.001 \| 0.001 \| \| COD (mg/L) \| In (SD) \| 216 (79.83) \| \| \| \| Out (SD) \| 81.20 (32.11) \| 75.73 (27.10) \| 79.6 (29.0) \| \| *p*-value \| 0.001 \| 0.001 \| 0.001 \| \| Turbidity (NTU) \| In (SD) \| 44.69 (32.42) \| \| \| \| Out (SD) \| 15.13 (13.58) \| 14.18 (9.33) \| 15.07 (12.27) \| \| *p*-value \| 0.001 \| 0.001 \| 0.001 \| \| TSS (mg/L) \| In (SD) \| 163.07 (82.67) \| \| \| \| Out (SD) \| 76.37 (49.70) \| 60.3 (30.53) \| 64.9 (36.35) \| \| *p*-value \| 0.001 \| 0.001 \| 0.001 \| \| Total Phosphorus (mg/L) \| In (SD) \| 11.15 (5.18) \| \| \| \| Out (SD) \| 4.61 (1.40) \| 4.48 (1.23) \| 5.00 (1.17) \| \| *p*-value \| 0.001 \| 0.001 \| 0.001 \| \| Nitrate – nitrogen (mg/L) \| In (SD) \| 27.3 (8.90) \| \| \| \| Out (SD) \| 18.23 (5.20) \| 18.29 (4.93) \| 20.32 (5.15) \| \| *p*-value \| 0.001 \| 0.001 \| 0.001 \| \| Total Coliforms (CFU/100 mL)** \| In (Median, IQR) \| 1457113 (1350000, 2370000) \| \| \| \| Out (Median, IQR) \| 66584 (13350,  152355) \| 63391 (20590, 265660) \| 108623 (26050,  109570) \| \| *p*-value \| 0.001 \| 0.001 \| 0.001 \| \| Fecal Coliforms (CFU/100 mL)** \| In (Median, IQR) \| 819206 (740000, 1186000) \| \| \| \| Out (Median IQR) \| 25991 (4630,  58840) \| 29676 (6470,  124227) \| 44720 (4200,  41515) \| \| *p*-value \| 0.001 \| 0.001 \| 0.001 \|   *In – influent stream; Out – effluent stream; SD – standard deviation; **Wilcoxon signed rank test; IQR – Interquartile range |
| --- | --- | --- | --- | --- | --- | --- | --- | --- | --- | --- | --- | --- | --- | --- | --- | --- | --- | --- | --- | --- | --- | --- | --- | --- | --- | --- | --- | --- | --- | --- | --- | --- | --- | --- | --- | --- | --- | --- | --- | --- | --- | --- | --- | --- | --- | --- | --- | --- | --- | --- | --- | --- | --- | --- | --- | --- | --- | --- | --- | --- | --- | --- | --- | --- | --- | --- | --- | --- | --- | --- | --- | --- | --- | --- | --- | --- | --- | --- | --- | --- | --- | --- | --- | --- | --- | --- | --- | --- | --- | --- | --- | --- | --- | --- | --- | --- | --- | --- | --- | --- | --- | --- | --- | --- | --- | --- | --- | --- | --- | --- | --- | --- | --- | --- | --- | --- | --- | --- | --- | --- | --- | --- | --- | --- | --- |
|  |
